# Raman Spectroscopy of Cell-Free Cervicovaginal Lavage for HPV Lesion Diagnosis: A Pilot Study

**DOI:** 10.3390/ijms262211064

**Published:** 2025-11-15

**Authors:** Elena Rimskaya, Alexey Gorevoy, Anastasia Devyatkina, Niso Nazarova, Natalia Starodubtseva, Patimat Abakarova, Anna Mgeryan, Sergey Kudryashov, Vera Prilepskaya, Gennady Sukhikh

**Affiliations:** 1 V.I. Kulakov National Medical Research Center for Obstetrics Gynecology and Perinatology, Ministry of Healthcare of Russian Federation, 117997 Moscow, Russia; 2 Moscow Center for Advanced Studies, 123592 Moscow, Russia; 3 Department of Obstetrics, Gynecology, Perinatology and Reproductology, Institute of Professional Education, Federal State Autonomous Educational Institution of Higher Education I.M. Sechenov First Moscow State Medical University of the Ministry of Health of the Russian Federation, 119991 Moscow, Russia

**Keywords:** Raman spectroscopy, cell-free CVL, HPV, cervical cancer, non-invasive diagnostics

## Abstract

High-risk human papillomavirus (HPV) is the leading etiological factor in cervical cancer, creating a pressing need for less invasive and more objective diagnostic tools. This pilot study pioneers the application of Raman spectroscopy to cell-free cervicovaginal lavage (CVL) for distinguishing between low-grade and high-grade squamous intraepithelial lesions (LSIL and HSIL) in HPV-positive patients. Raman spectra were acquired at 532-nm excitation from cell-free CVL samples of 20 patients with histologically confirmed LSIL (*n* = 9) or HSIL (*n* = 11). Comparative analysis of Raman bands revealed a significant biochemical shift in HSIL, presumably characterized by reduced glycogen and lactate/lactic acid levels alongside substantially elevated heme proteins. A diagnostic model based on key spectral intensity ratios achieved differentiation between LSIL and HSIL with 80% sensitivity and 86% specificity. These findings demonstrate that Raman spectroscopy of cell-free CVL effectively captures profound metabolic and microvascular alterations characteristic of neoplastic progression, showcasing its strong potential as a rapid, cost-effective, non-invasive, and objective tool for cervical lesion risk stratification.

## 1. Introduction

Cervical diseases associated with human papillomavirus (HPV) are a major challenge in onco-gynecology [1,2]. In 2022, cervical cancer ranked fourth globally in both incidence and mortality among women, with 662,000 new cases and 349,000 deaths [3]. High-risk HPV is the leading cause, responsible for >95% of cervical cancers, 70% of vulvar/vaginal cancers, 60% of oropharyngeal cancers, and 90% of anal canal cancers [1,4].

The most widely applied and standardized method of cervical cancer screening is cytology followed by histological examination for the final confirmation of the diagnosis. At the same time, this is an invasive, labor-intensive, and time-delayed procedure, which exhibits significant limitations related to lesion visibility and the risk of missing altered areas [5,6]. While colposcopy aids in visual diagnosis and biopsy targeting, its accuracy is operator-dependent and lacks molecular insight [7]. This creates a need for new, sensitive, and accessible techniques. HPV DNA testing is now the primary screening method due to its high sensitivity for precancer [8]. Optical methods like fluorescent and multispectral imaging, optical coherence tomography, and Raman spectroscopy can expand ex vivo and in vivo analysis by providing a biochemical basis for visual assessment [2,9,10]. Analyzing biofluids via liquid chromatography-mass spectrometry (LC-MS), nuclear magnetic resonance (NMR), or spectroscopy for proteomic/metabolomic markers is a promising non-invasive approach [11,12].

Raman spectroscopy is a rapid, contact-free, and non-destructive universal method for analysis of biomedical samples, such as cells, tissues, and fluids, identifying specific molecular vibrations from various biochemical components, including DNA, RNA, proteins, lipids, and carbohydrates. It shows great potential for noninvasive detection of cervical precancer and cancer in cells and tissues, with studies reporting accuracies up to 98.5% for distinguishing HPV status and 90% sensitivity for identifying high-grade squamous intraepithelial lesions (HSIL) [13,14,15,16,17,18]. Beyond cells and tissues, cervicovaginal fluid (CVF), containing epithelial cells of the cervix and vagina, and intercellular and drainage fluids, can provide a broader picture of pathological changes. Initially used forensically [19,20,21], Raman spectroscopy of dried cell-free CVF has also been explored for detecting HPV and dysplasia, though without reported classification rates [1,22]. Cell-free CVF offers more stable Raman signals by eliminating interference from cellular debris, bacteria, and mucus.

This work uses cell-free cervicovaginal lavage (CVL) for its non-invasive, standardized collection, overcoming CVF’s reproducibility issues. We employed Raman spectroscopy to analyze cell-free CVL, identifying biomarkers to distinguish between HPV-associated low- and high-grade squamous intraepithelial lesions (LSIL and HSIL) with high classification rates, demonstrating its potential for predicting neoplastic transformation risk.

## 2. Results

### 2.1. Cell-Free Cervicovaginal Lavage Composition Revealed by Spectral Analysis

To investigate the biochemical composition of cell-free CVL, we applied multivariate curve resolution (MCR) analysis [23,24,25] to the processed Raman spectra from a set of CVL samples (n=20) obtained from patients with histologically confirmed LSIL or HSIL and presence of HPV. This method decomposes the spectral data into non-negative components representing the spectral profiles of constituents and their relative weights (scores), thereby facilitating biochemical interpretation. Although applying this deconvolution to Raman spectra rarely allows unambiguous identification of pure chemical substances, this method is still effective for revealing correlations in intensities of Raman bands further used for differentiation and highlighting their probable assignments.

**Figure 1:**
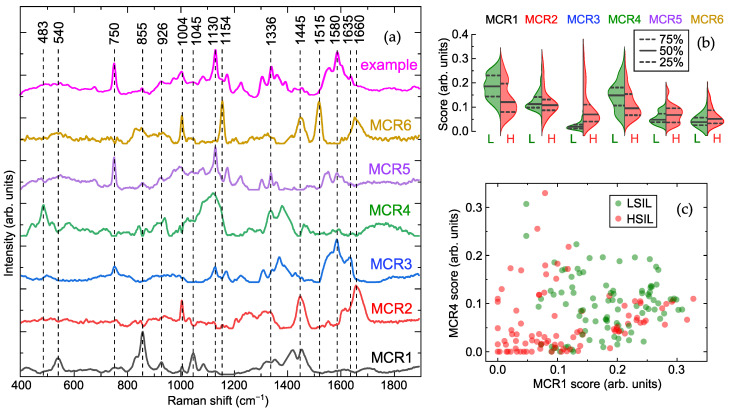
Results of multivariate curve resolution (MCR) analysis: (**a**) example of acquired Raman spectrum and six components obtained by MCR analysis with their major Raman bands, (**b**) half-violin plots indicating scores of these components for low- (LSIL, L) and high-grade squamous intraepithelial lesion (HSIL, H) samples with median (50%) and quartile (25%, 75%) levels, and (**c**) distribution of scores for first (MCR1) and fourth (MCR4) components.

Figure 1a demonstrates six major components resolved from the Raman spectra acquired at 532-nm excitation. Based on comparisons with our database and literature [19,26], the characteristic bands of MCR1 at 540, 830, 855, 926, 1045, 1088, 1420, and 1455 ${\mathrm{cm}}^{-1}$ coincide with the main bands in the spectra of lactate/lactic acid. Possible correspondences of Raman bands and functional groups are listed in Table S1, and a comparison of the spectral profiles of the MCR components with Raman spectra from the literature is presented in Figures S1–S4 in Supplementary Materials. Next, the spectral composition of MCR2 and MCR6 is close to the typical Raman spectra of proteins, featuring the aromatic ring breathing mode and Amide bands at 1004, 1445, and 1660 ${\mathrm{cm}}^{-1}$ [19,27,28]; however, MCR6 also demonstrates prominent carotenoid bands at 1004, 1154, and 1515 ${\mathrm{cm}}^{-1}$ [29,30,31] and may additionally be affected by lipoprotein content [32]. Many vibration bands presented in MCR3 and MCR5 can be associated with heme proteins (e.g., hemoglobin and myoglobin), namely 750, 1130, 1225, 1305, 1336, 1360, 1555, 1585, and 1635 ${\mathrm{cm}}^{-1}$ [33,34]. Finally, MCR4 can be tentatively identified as glycogen, showing related bands at 483, 575, 853, 940, 1080, 1128, 1336, 1382, and 1457 ${\mathrm{cm}}^{-1}$ [14,35,36].

Analysis of the component scores (Figure 1b) revealed elevated levels of MCR3 and MCR5 (heme proteins) in most HSIL samples, which were low or absent in LSIL. Additionally, MCR1 (lactate/lactic acid) and MCR4 (glycogen) were markedly reduced in the HSIL group compared to LSIL, while MCR2 content remained consistent across the groups. The distribution of MCR1 ( lactate/lactic acid) and MCR4 (glycogen) scores (Figure 1c) showed a clear separation between LSIL and HSIL. Using quadratic discriminant analysis (QDA) on these two components, differentiation between LSIL and HSIL achieved a sensitivity of 60% and specificity of 78% for HSIL detection (for the test set; corresponding values for the training set were 61% and 80%). Incorporating heme-related components (MCR3 and MCR5) increased specificity to 88% while maintaining sensitivity at 62% (93% and 63% for the training set). However, further improving sensitivity without substantial loss of specificity remains challenging. Despite the effective identification of spectral differences between the groups, the hypothetical nature of the proposed biochemical interpretations should be emphasized.

### 2.2. Optimal Spectral Ratios for Differentiating Cervical Lesions via Raman Spectroscopy

To identify optimal spectral criteria for differentiating between LSIL and HSIL samples, we analyzed all detected Raman bands individually, representing each spectrum as an array *I* of its peak intensities. Each band was iteratively treated as a reference (see Section 4), and all possible pairs of intensity ratios were examined. Receiver operating characteristic (ROC) curves for the QDA classifier were computed for each pair to compare specificity at a target sensitivity. The most robust classification was achieved using $I(1004\;{\mathrm{cm}}^{-1})$ as the denominator in all ratios, as this band–associated with aromatic ring breathing of phenylalanine in proteins [27]–served as a stable reference due to consistent protein content across both sample groups.

**Figure 2:**
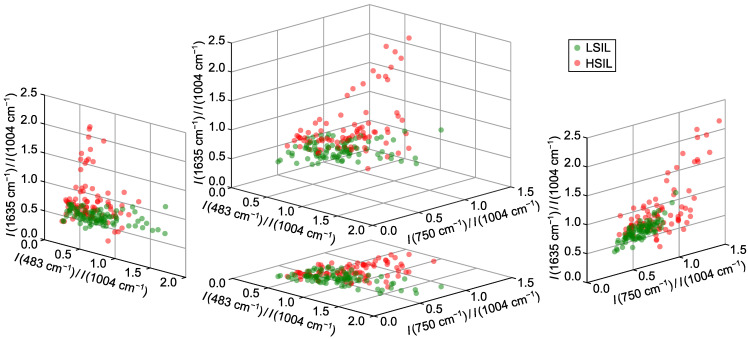
Distributions of normalized intensity values (intensity ratios) for three selected Raman bands at 532 nm excitation wavelength providing differentiation between LSIL and HSIL samples.

According to the results of the tests, three best spectral ratios were identified as $I\left(483\;{\mathrm{cm}}^{-1}\right)/I\left(1004\;{\mathrm{cm}}^{-1}\right)$, $I\left(750\;{\mathrm{cm}}^{-1}\right)/I\left(1004\;{\mathrm{cm}}^{-1}\right)$ and $I\left(1635\;{\mathrm{cm}}^{-1}\right)/I\left(1004\;{\mathrm{cm}}^{-1}\right)$. These ratios collectively provided an overall true positive rate (TPR) of 82%, with a specificity of 86% (90% for the training set) and sensitivity of 80% (fixed value). The peak at 483 ${\mathrm{cm}}^{-1}$, present in the glycogen-associated MCR4 component [14,36,37,38], resulted in a higher ratio for LSIL samples, as confirmed by distribution analysis (Figure 2). The band at 750 ${\mathrm{cm}}^{-1}$, linked to the CH–N–C breathing stretch in porphyrin rings, served as a blood marker in HSIL samples [39]. The ratio $I\left(1635\;{\mathrm{cm}}^{-1}\right)/I\left(1004\;{\mathrm{cm}}^{-1}\right)$ was primarily influenced by heme proteins (band at 1635 ${\mathrm{cm}}^{-1}$ [33]), with minimal contribution from the Amide I protein band [40] due to normalization at 1004 ${\mathrm{cm}}^{-1}$.

Classification was driven mainly by the bands at 750 and 1635 ${\mathrm{cm}}^{-1}$, which showed increased intensity in HSIL samples and alone achieved 75% specificity at 80% sensitivity. Incorporating $I\left(483\;{\mathrm{cm}}^{-1}\right)/I\left(1004\;{\mathrm{cm}}^{-1}\right)$ improved results by distinguishing HSIL samples with low heme content from LSIL via reduced glycogen levels; comparable results were observed for other glycogen or lactate bands. Using more than three ratios did not yield significant improvements, confirming the optimality of the selected criteria.

## 3. Discussion

This study pioneers the application of Raman spectroscopy to cell-free CVL for distinguishing between LSIL and HSIL in patients with HPV infection. Using MCR analysis and further validation through the analysis of specific Raman bands, we indicated key spectral differences between the groups of samples and associated them with the possible constituents of CVL identified as lactate/lactic acid, general proteins, heme proteins and glycogen. This approach aligns with and significantly expands upon earlier forensic-oriented work by Sikirzhytskaya et al. [19], who used factor analysis to decompose Raman spectra of vaginal fluid into similar constituents, including lactic acid and proteins, yet did not explore its diagnostic potential for cervical precancer.

Our comparative analysis revealed statistically significant biochemical shifts between LSIL and HSIL groups: the characteristic Raman peaks of glycogen and lactate/lactic acid were markedly reduced in HSIL samples, while the spectral features of heme proteins were substantially more intense. These changes reflect profound metabolic and microenvironmental alterations during cervical lesion progression. The observed glycogen depletion is clinically significant, as glycogen in healthy cervical epithelium supports a protective vaginal microbiome by fueling lactobacilli-produced lactic acid [41]. Its reduction in HSIL aligns with metabolic reprogramming, where glycogen is mobilized to fuel biosynthetic pathways [14]. Similar to processes in immune cells during inflammation, glycogen breakdown in precancerous cells supplies the pentose phosphate pathway for NADPH production, supporting proliferation, redox balance, and chemoresistance [42]. This reprogramming is a hallmark of the Warburg effect, with HPV-associated lesions exhibiting heightened glycolysis despite oxygen availability to meet energetic and anabolic demands [14,43,44,45].

Paradoxically, despite enhanced glycolysis, HSIL samples showed decreased lactate/lactic acid levels. This contrasts with typical cancer metabolism, where glycolytic flux increases lactate production and acidifies the microenvironment [45,46,47]. This discrepancy may be explained by the reverse Warburg effect [14,48], wherein cancer-associated stromal fibroblasts undergo aerobic glycolysis and export lactate, which is then imported by epithelial cancer cells via monocarboxylate transporters (e.g., MCT1) to fuel anabolic pathways [49]. Cancer cells can utilize lactate directly in the tricarboxylic acid (TCA) cycle, demonstrating metabolic flexibility beyond glucose dependence [50,51,52]. Alternatively, reduced lactate may indicate a shift toward glutaminolysis, with glutamine replenishing TCA cycle intermediates under mitochondrial dysfunction [53,54].

The prominent presence of heme proteins in HSIL samples, evidenced by their characteristic bands (e.g., 750 and 1635 ${\mathrm{cm}}^{-1}$), reflects the vascular nature of neoplastic tissues and associated microhemorrhages [11,18]. This aligns with previous studies detecting hemoglobin derivatives in biofluids as cancer markers [55]. These signals indicate pathological angiogenesis driven by vascular endothelial growth factor (VEGF) and other pro-angiogenic factors [55,56,57]. As dysplasia advances, destruction of the basement membrane and tissue architecture increases fragility and bleeding risk [58]. Thus, heme detection in CVL via Raman spectroscopy serves not merely as an indicator of contamination but as a valuable non-invasive biomarker of active angiogenesis and structural disruption, signaling lesion progression and invasive potential.

We should note that the performed MCR analysis of the acquired Raman spectral data was aimed at detecting spectral differences between the samples and establishing their possible biochemical interpretation, rather than at identifying the exact chemical composition of the samples. The latter task requires validation using other methods of chemometric analysis and preparation of control samples with known concentrations of components for Raman measurements to develop a biochemical model of cell-free CVL and test its photochemical stability, which can be an important step towards the standardization of the proposed technique for integration into clinical practice.

The findings of our analysis were used to create a diagnostic model based on specific Raman peak intensity ratios. Effective differentiation between LSIL and HSIL was achieved using the ratios $I\left(483\;{\mathrm{cm}}^{-1}\right)/I\left(1004\;{\mathrm{cm}}^{-1}\right)$, $I\left(750\;{\mathrm{cm}}^{-1}\right)/I\left(1004\;{\mathrm{cm}}^{-1}\right)$, and $I\left(1635\;{\mathrm{cm}}^{-1}\right)/I\left(1004\;{\mathrm{cm}}^{-1}\right)$, which leverage the metabolic and vascular changes described. The successful application of this strategy supports the potential of Raman spectroscopy as a complementary tool in cervical cancer screening, offering a rapid, non-invasive, cost-effective method to assess lesion severity based on underlying biochemical alterations.

These promising results must be interpreted within the study’s limitations, including a small sample size of only 20 patients, which limits statistical power and generalizability, the pilot design with potential selection bias, and the cross-sectional nature that cannot establish causality or predict progression from LSIL to HSIL over time. The performance metrics, while highly encouraging, require further confirmation with a larger set of samples, including double-blind testing for truly unseen data. While sample preparation minimizes cellular debris, confounding factors in the cervicovaginal microenvironment—such as microbiome variations or non-specific inflammation—may influence Raman spectra and require further investigation. To advance these findings, future work should validate the methodology in a larger, multi-center prospective cohort, include control groups of HPV-negative and HPV-positive women with normal cytology to define the full diagnostic range and triage capability, and explore correlations between Raman biomarkers and proteomic or metabolomic profiles to deepen molecular understanding. Technologically, efforts should streamline the protocol and develop automated, portable Raman systems for potential point-of-care use, with the goal of integrating the approach into existing cervical cancer screening algorithms.

## 4. Materials and Methods

### 4.1. Sample Collection

The study included 20 women with a mean age of 30.55±7.6 years (median 28 years; range 22–49) who were treated at the scientific and outpatient department of the National Medical Research Center for Obstetrics, Gynecology and Perinatology named after Academician V.I. Kulakov (Moscow, Russia) from January to March 2025. Inclusion criteria comprised reproductive age from 20 to 49 years, a regular menstrual cycle, histologically confirmed LSIL (CIN I) or HSIL (CIN II/III) with the presence of carcinogenic risk HPV, and an ability to comply with protocol requirements. Exclusion criteria were pregnancy, lactation, hormone therapy, acute inflammation, decompensated dysfunction of the kidneys, liver, or lungs, and psychoneurological conditions. The sample collection was preferentially scheduled during the follicular phase of the menstrual cycle. Based on histological examination of biopsy material, two groups were formed: LSIL (n=9) and HSIL (n=11).

CVL samples were collected into 15 mL Falcon tubes after irrigating the vagina and cervix with a 5 mg/mL solution of sodium chloride 0.9% prior to routine procedures (biopsy for histological examination, etc.) to minimize blood contamination. Samples were centrifuged at 2000× *g* for 10 minutes at 4 °C. The resulting supernatant was aliquoted into three cryovials, each containing 1.5 mL of liquid, and frozen at −80 °C. Total processing and storage preparation time was under 30 min, with frozen samples stable for up to 3 years, preserving the biochemical composition for reliable Raman analysis [20,59,60,61,62]. Wide-spectrum HPV genotyping for 21 HPV types was performed using real-time polymerase chain reaction developed by DNK-Technologia (Moscow, Russia).

Extended colposcopy was performed using a Leisegang colposcope (Leisegang, Berlin, Germany) following the International Federation for Cervical Pathology and Colposcopy (IFCPC) Terminology (Rio de Janeiro, 2017) [63]. Histological verification used a two-level classification where mild epithelial dysplasia (CIN I) corresponds to LSIL, and moderate/severe cervical intraepithelial neoplasia (CIN II/III) refers to HSIL.

### 4.2. Raman Spectroscopy

Before the spectral measurements, the CVL samples were allowed to thaw at room temperature (25 °C). A small aliquot of each sample (5–10 µL) was deposited onto a glass slide coated with aluminum and permitted to dry completely, which is a preferential setup for intense Raman signal with lower background fluorescence [64]. The spectra were acquired with a Confotec MR520 confocal microscope-spectrometer (SOL Instruments, Minsk, Belarus) using 532 nm laser excitation (20 mW power in the sample plane and a maximum accumulation time of 10 s) and a 40× objective lens MPlanFL (Nikon, Tokyo, Japan) with a numerical aperture of 0.75. The spectral profiles remained stable for different exposure times (1–10 s), which indicated photochemical stability and repeatability under the given measurement conditions. According to the specifications provided by the manufacturers, the spectral resolution was in the range from 1 to 1.5 ${\mathrm{cm}}^{-1}$. In total, 15–20 spectra were obtained at random points of each sample at room temperature under equal conditions.

### 4.3. Raman Data Processing and Analysis

The acquired spectra were preprocessed using the MATLAB (R2022b, MathWorks, Natick, MA, USA) implementations of the Vancouver Raman Algorithm [31,65,66] to remove fluorescence background with a modified multi-polynomial baseline fitting and the Savitzky–Golay filter to reduce noise. Subsequently, MCR analysis [23,24,25] was performed via a non-negative matrix factorization algorithm with an alternating least squares (ALS) approach (built-in MATLAB functions). In our study, the MCR-ALS analysis was applied to the processed Raman spectra of all CVL samples normalized by their mean value, and the optimal number of interpretable components was identified as six by means of monitoring a decrease in residual error with increasing number of components and checking the results for duplicates. Possible assignments for each MCR component and its major spectral bands were found using comparison with Open Raman spectral library [36], our collected Raman spectra database of biomolecules, and data from various literature sources.

To evaluate the suitability of various spectral features for distinguishing CVL samples as LSIL vs. HSIL, we replaced each spectrum with an array *I* of its peak intensities (e.g., $I(483\;{\mathrm{cm}}^{-1})$) in the main Raman bands, the list of which was formed as a result of the aforementioned analysis. For the calculation of the classification rates, we sequentially tested all possible normalizations by selecting one band as the reference, for example $I(1004\;{\mathrm{cm}}^{-1})$, and dividing the intensities of the other bands by it to form an array of ratios, such as $I\left(483\;{\mathrm{cm}}^{-1}\right)/I\left(1004\;{\mathrm{cm}}^{-1}\right)$, $I\left(750\;{\mathrm{cm}}^{-1}\right)/I\left(1004\;{\mathrm{cm}}^{-1}\right)$, and so on [25]. Next, we used the QDA in MATLAB Classification Learner with default settings to compute the ROC curve for each pair of ratios in the array and evaluate the specificity at 80% sensitivity as the criterion for selecting the best pair of ratios. The results presented in the article correspond to the average values for the test set (or the training set, if specified) of 5-fold stratified cross-validation with 10 repetitions to avoid model overfitting [67]. The spectra of each sample (belonging to one patient) were included into only one subset [68]. All data processing algorithms were implemented by the authors as custom MATLAB scripts, unless otherwise stated.

## 5. Conclusions

In conclusion, this study establishes that Raman spectroscopy of CVL provides a powerful, non-invasive window into the profound biochemical transformations underpinning HPV-associated cervical carcinogenesis. The noticeable spectral differences can be interpreted as markers of pathophysiological changes—including glycogen depletion, altered lactate/lactic acid dynamics and microvascular alterations evidenced by elevated heme proteins—which collectively form a composite signature of neoplastic progression. This approach demonstrates high sensitivity and specificity in distinguishing HSIL from LSIL (80% and 86%, respectively), showcasing its strong potential to overcome the key limitations of conventional diagnostics, namely the subjectivity of cytology and the invasiveness of biopsy. By offering a rapid and objective molecular assessment, Raman spectroscopy of CVL could significantly enhance risk stratification and facilitate personalized clinical management. While these pilot findings are highly promising, their translation into clinical practice necessitates further validation through larger, prospective multi-center studies to confirm efficacy and integrate this innovative methodology into standardized screening algorithms.

## Data Availability

The data presented in this study are available on request from the corresponding author. The data are not publicly available due to privacy.

## Supplementary Materials

The following supporting information can be downloaded at: https://www.mdpi.com/article/10.3390/ijms262211064/s1. References [14,19,26,27,29,33,34,35,36,69,70,71,72,73,74,75,76,77,78,79,80,81,82,83,84,85,86,87,88,89,90,91,92,93,94] are cited in the Supplementary Materials.
