# Supplementary material for: Raman Spectroscopy of Cell-Free Cervicovaginal Lavage for HPV Lesion Diagnosis: A Pilot Study
Source: Int J Mol Sci. 2025 Nov 15;26(22):11064. doi: 10.3390/ijms262211064 (PMC12652234; doi:10.3390/ijms262211064)
Supplement: Supplementary file 1 [file ijms-26-11064-s001.zip › ijms-3941964-supplementary.pdf]

Supplementary Materials: Raman Spectroscopy of Cell-Free Cervicovaginal Lavage for HPV Lesion Diagnosis: A Pilot Study

Table S1 lists possible assignments for the main Raman bands detected in six major components resolved from the Raman spectra acquired at 532-nm excitation through MCR analysis (see also Figure 1a in the main text), including corresponding functional groups and relevant references. For each MCR component, we analyzed band positions and potentially associated functional groups, proposing biochemical interpretations based on the coincidence of multiple bands. Consequently, we validated the multi-band correlation by comparing the MCR spectral profiles with matching Raman spectra from the literature, as shown in Figures S1, S2, S3, and S4. Since the MCR components can represent mixtures of constituents from a broad class (e.g., heme proteins) with similar functional groups, some of the compared spectra exhibit different band intensity ratios and should be considered as representative examples of the associated class.

Thus, the characteristic bands of MCR1 at 540, 830, 855, 926, 1045, 1088, 1420, and 1455 cm^{-1} along with less-pronounced non-indicated bands coincide with the main features in the spectra of lactate/lactic acid (see Figure S1). The spectra of other candidates demonstrate both matching (e.g., 830, 855, 926, 1045, 1455 cm^{-1} for proline) and non-matching bands, generally providing a lower correlation score.

Next, the spectral composition of MCR2 and MCR6 is close to the typical Raman spectra of proteins, featuring the aromatic ring breathing mode and Amide bands at 1004, 1445, and 1660 cm^{-1} (see Figure S2). The Raman spectra of major proteinase, keratin, and albumin are given in Figure S2 as examples of proteins. MCR6 also demonstrates prominent carotenoid bands at 1004, 1154, and 1515 cm^{-1} and may actually represent lipoproteins due to the known overlap of lipid and protein bands around 1445 and 1660 cm^{-1}.

Many vibration bands presented in MCR3 and MCR5 can be associated with heme proteins (e.g., hemoglobin and myoglobin), namely 750, 1130, 1225, 1305, 1336, 1358, 1370, 1555, 1585, 1635 cm^{-1}, and some others (see Figure S3). Some differences in the intensity of closely positioned bands (e.g., 1336 and 1358 cm^{-1}) can presumably reflect transitions between the two states of Fe atoms in heme proteins.

Finally, MCR4 can be tentatively identified as glycogen, showing major related bands at 483, 575, 853, 940, 1080, 1128, 1336, 1382, and 1457 cm^{-1}, as well as a similar spectral profile (see Figure S4).

Table S1. Possible assignments of main Raman bands.

Position, cm^{-1}	Band assignments	Components	References
483	glycogen	glycogen	[1–10]
540	$\delta(C_3CO)$, $\delta(C-O-O)$ deformation; $\delta(C-O)$, glucose-saccharide band $\delta(C_2—C_1—O)$; $\rho(CO_2)$	lactate/lactic acid, glucose	[7,8,11–14]
575	ν(C—C)	glycogen, tryptophan (Trp)	[1–9]
750	ν_{15}/ν (pyr breathing)	heme proteins	[15–17]
830	out-of-plane ring breathing, $\nu_2(PO_2^-)$ stretch of nucleic acids, (C—H) out of plane bending in benzoid ring	lactate/lactic acid, glucose, proline (Pro), hydroxyproline, tyrosine (Tyr)	[3,7,8,11–13]
853	(C—C) stretching	glycogen	[1–10]
855	(C—C) stretch of Pro ring; (C—O—C) skeletal mode; ν(C—C), Pro + δ(CCH) ring breathing; $\nu(C—C_N)$ symmetric stretching vibration; Tyr (protein assignment and polysaccharide); carbohydrates (glycogen, polysaccharides)	Pro, Tyr, lactate/lactic acid, glycogen	[3,7,8,10–14,18]
926	(C—C) stretch of Pro ring/glucose/lactic acid; ν(C—C), stretching-probably in amino acids	lactate/lactic acid, Pro, glucose	[3,7,8,11,12]
940	(C—C) stretching; skeletal modes (polysaccharides)	glycogen	[1–10]
1004	ν(C—C) aromatic ring breathing, phenylalanine (Phe); $\rho(CH_3)$, carotenoids	proteins (Phe), collagen, elastin, keratin, carotenoids	[3,19–24]
1045	carbohydrates peak for solutions and solids, Pro (collagen assignment), symmetric stretching vibration of $\nu_3(PO_4^{3-})$	lactate/lactic acid, Pro	[3,7,8,11,12,14]
1080	phosphate vibrations (phosphodiester groups in nucleic acids); collagen	glycogen, phospholipids	[1–9]
1088	symmetric (PO_2^-) stretching, (C—C) stretch	lactate/lactic acid	[3,7,11,12,14]
1128	CH_3 rocking and (C—O) stretching (carbohydrates), ν(C—O) + ν(C—C), disaccharides	glycogen	[1–9]
1130	ν_{22}, ν_{14}	heme proteins	[1,15–17]
1155	ν(C—C) and ν(C—N)	carotenoids	[20,23–25]
1225	ν_{42}, ν_{13}, Amide III	heme proteins, Amide III	[1,15–17]
1305	ν_4, ν_{21}	heme proteins	[1,15–17]
1336	ν(C—N), β(N—H), ν(C—H), proteins; $\omega(CH_2, CH_3)$, nucleic acids; ν_{41}, heme; Amide III and CH_2 wagging vibrations from glycine backbone and Pro side chain	proteins (aliphatic AA, Trp), A, G, heme proteins, glycogen, Trp	[1,3,15–17,20,25–27]
1358	$\nu_4(Fe^{2+})$; ν(C—N), ν(C—H)	Trp, Tyr, heme proteins	[1,3,15–17,28,29]
1370	$\nu_4(Fe^{3+})$	heme proteins	[1,15–17,29]
1385	(CH_3) symmetric bending	glycogen, nucleoproteins	[1–10]
1420	(C—H) deformation (DNA/RNA and proteins and lipids and carbohydrates); (CH_2) bending mode of proteins and lipids	lactate/lactic acid, deoxyribose, aspartic acid, glutamic acid, fatty acids	[3,7,11,12,14]

Continued on the next page

Table S1 - *Continued*

**Position, cm^{-1}**	**Band assignments**	**Components**	**References**
1445	δ(C—H), proteins; $\alpha(CH_2, CH_3)$, lipids; cholesterol band; $\nu_{28}(C_\alpha C_m)$, observed in the spectra of single human RBC	proteins (aliphatic AA), keratin, collagen, elastin, lipids, phospholipids	[3,20,25,29–31]
1455	(CH_2) stretching/(CH_3) asymmetric deformation; overlapping asymmetric (CH_3) bending, (CH_2) scissoring (is associated with elastin, collagen, phospholipids)	lactate/lactic acid, collagen, phospholipids, deoxyribose	[3,7,11,12,14]
1457	(CH_2) asymmetric bending; (CH_3) asymmetric deformation	glycogen, deoxyribose	[1–10]
1515	ν(C=C)	carotenoids	[20,23,24,32]
1555	$\nu_4(Fe^{2+})$; ν(C—H)	Trp, heme proteins	[1,3,15–17,28,29]
1585	$\nu_{19}(Fe^{3+})$; pyrimidine ring (nucleic acids)	heme proteins	[1,3,15–17,28,29]
1615	(C=C) (protein)	Tyr, Trp	[3,29]
1635	$\nu_{10}(Fe^{3+})$; Amide I band	Amide I, heme proteins	[1,3,15–17,28,29]
1660	Amide I (α-helix), ν(C=O), proteins; ν(C=C), lipids; cholesterol band	proteins, collagen, elastin, keratin, lipids (unsaturated), triglycerides (fatty acids)	[3,20,25,29–31,33–35]

* Excitation wavelength. ** α, scissoring; β, bending; δ, deformation; ν, stretching; ρ, rocking; τ, twisting; ω, wagging. *** Aromatic amino acids (AA): Phe, phenylalanine; Trp, tryptophan; Tyr, tyrosine. Aliphatic AA: Pro, proline. Nucleic acids: A, adenine; C, cytosine; G, guanine, T, thymine.

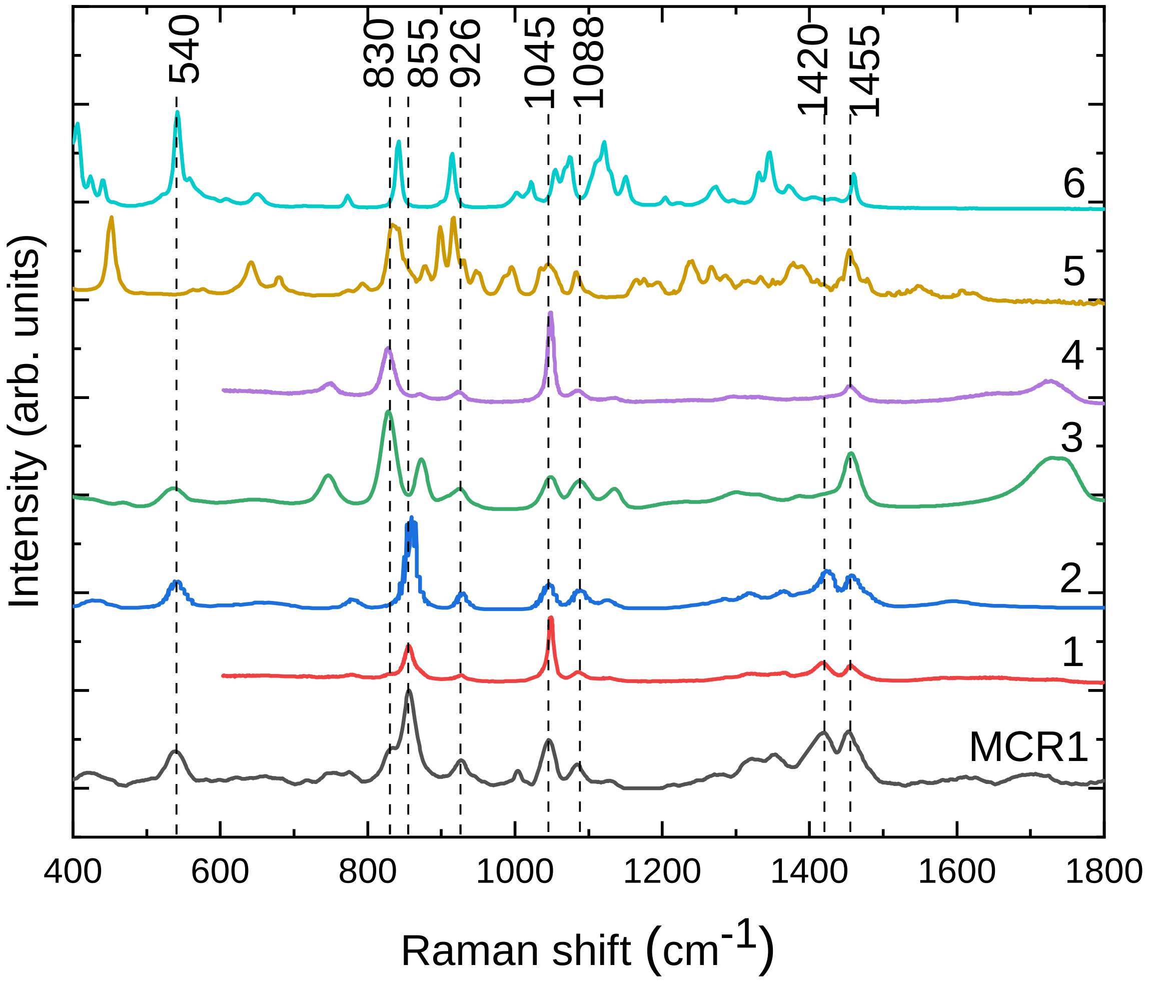

Figure S1. Comparison of MCR1 component with Raman spectra from other sources: 1 — lactate [7], 2 — lactate [36], 3 — lactic acid (90% purity, №L812422-100 ml, Macklin Biochemical Technology Co., Ltd. (Shanghai, China)) (own results), 4 — lactic acid [7], 5 — proline [13], and 6 — glucose [5].

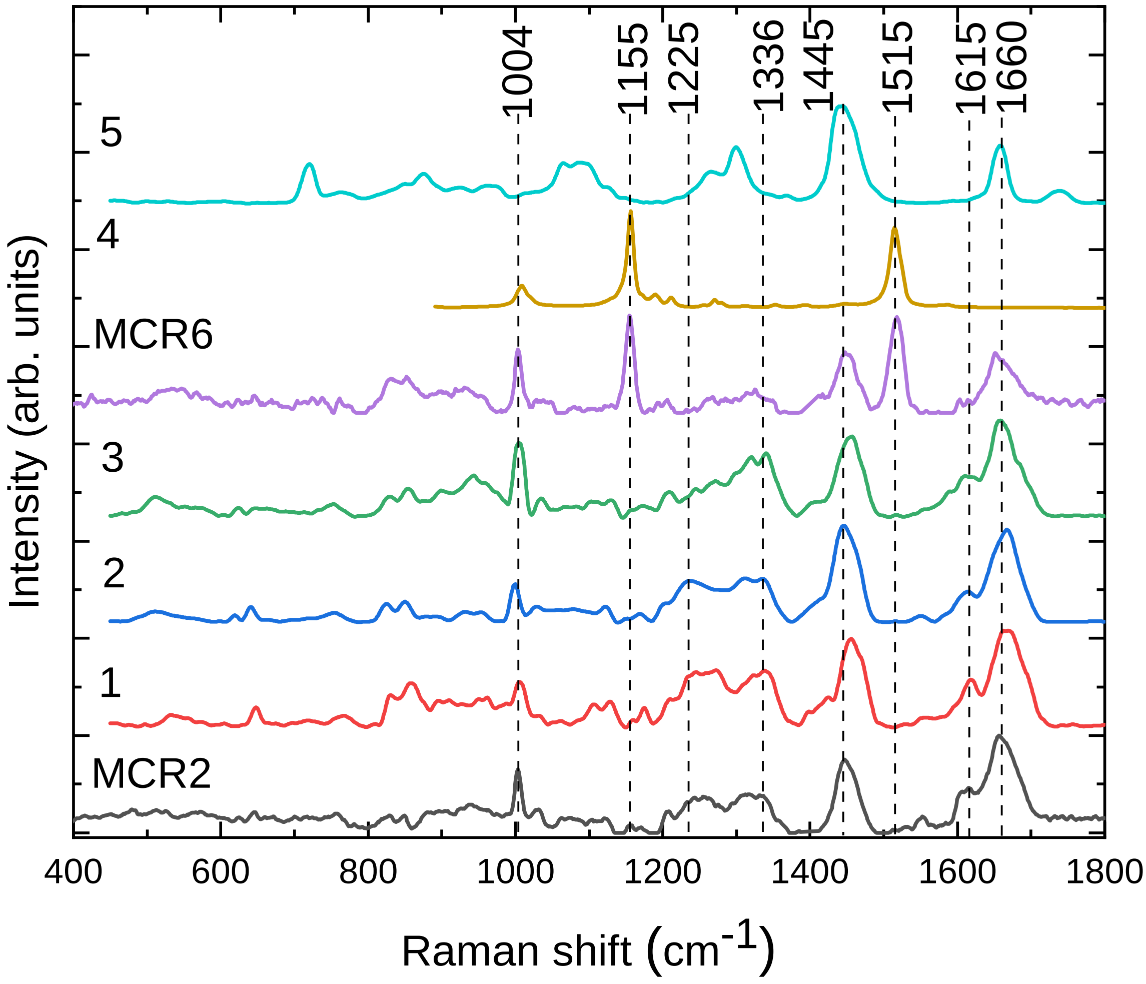

Figure S2. Comparison of MCR2 and MCR6 components with Raman spectra from other sources: 1 — major proteinase [6], 2 — keratin [6], 3 — albumin [6], 4 — *β*-carotene [24], 5 — phosphatidylcholine [6].

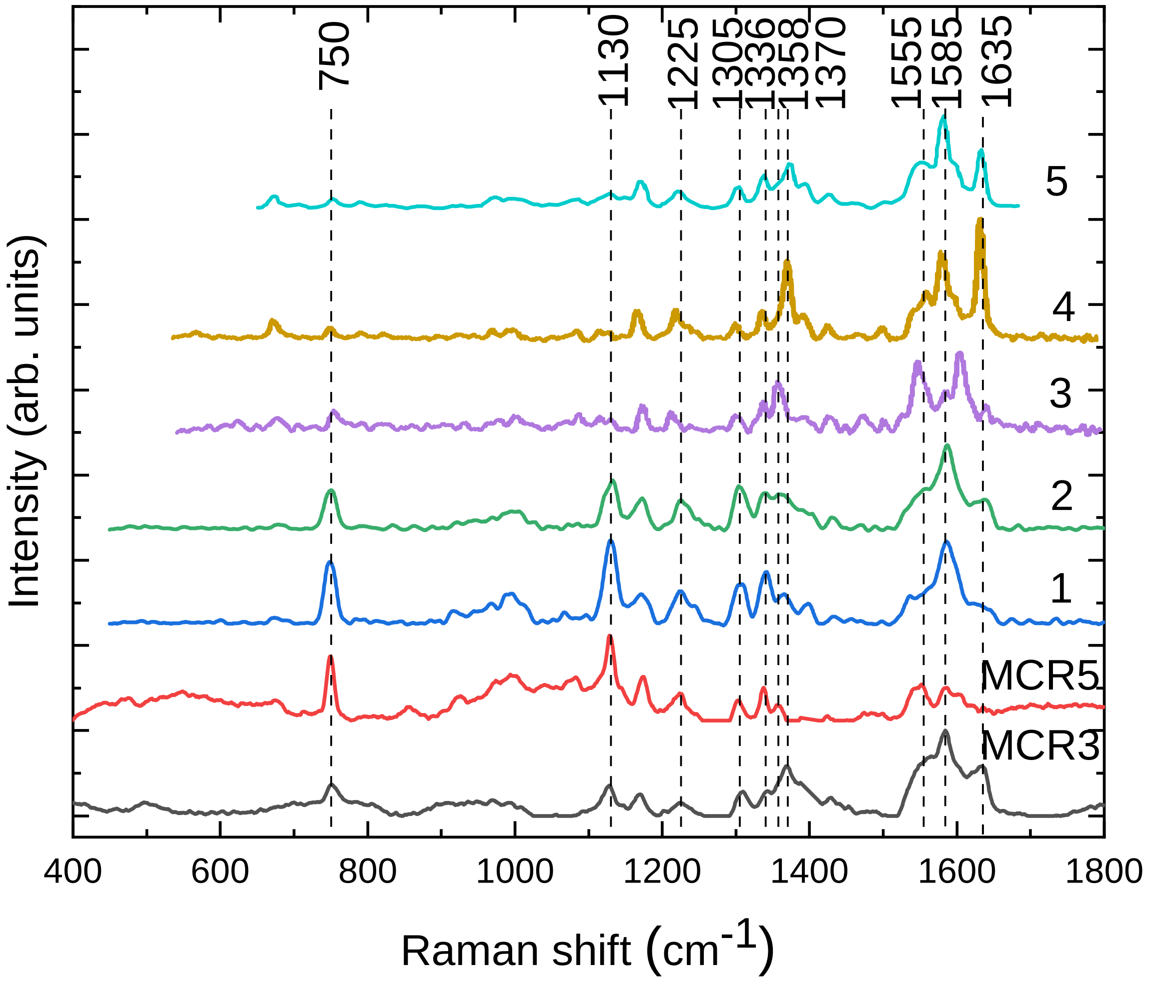

Figure S3. Comparison of MCR3 and MCR5 components with Raman spectra from other sources: 1 — hemoglobin [6], 2 — myoglobin [6], 3 — deoxyhemoglobin [17], 4 — oxyhemoglobin [17], 5 — oxyhemoglobin [15].

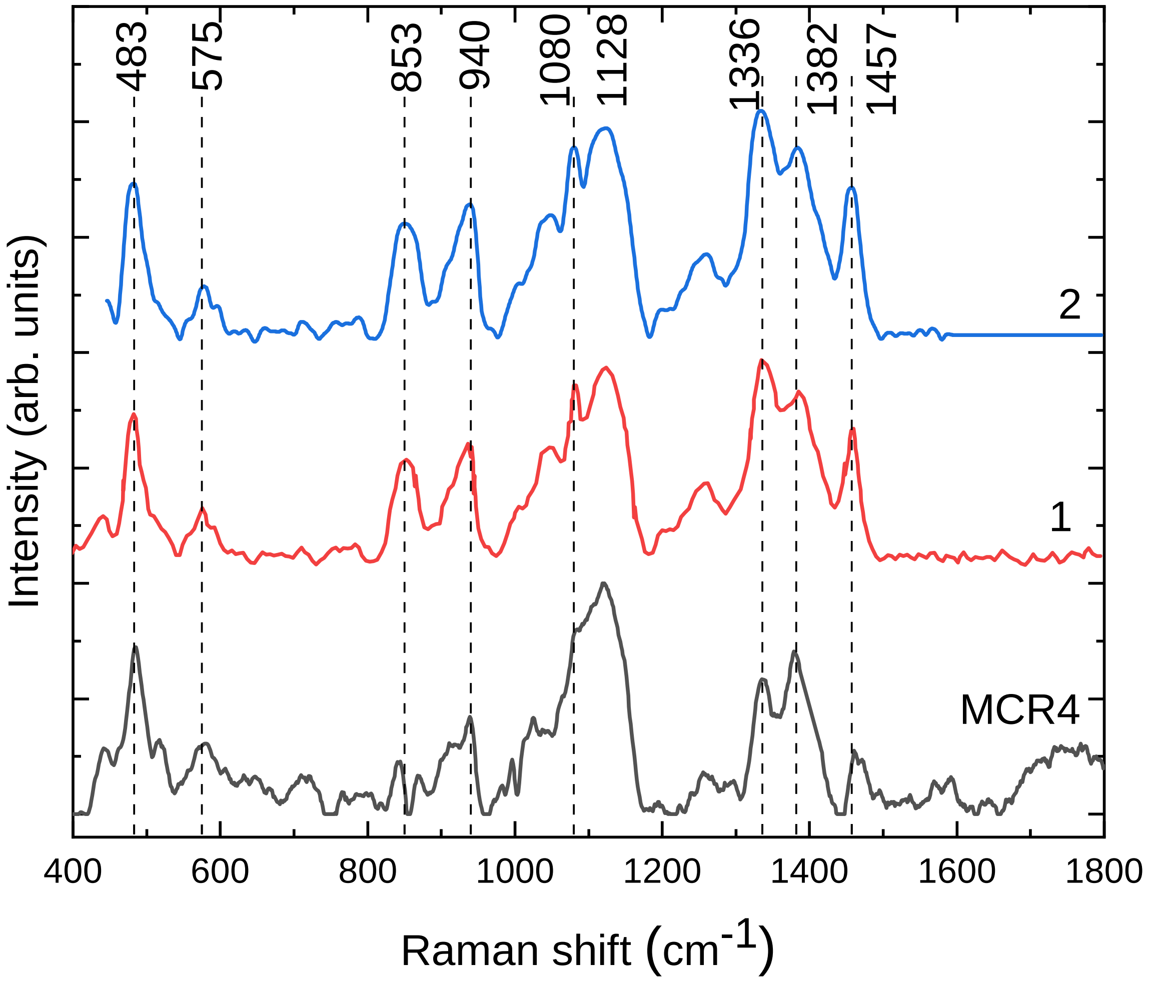

Figure S4. Comparison of MCR4 component with with Raman spectra from other sources: 1 — glycogen [4], 2 — glycogen [6].

References

1. Adamczyk, A.; Tipping, W.; Mazuryk, O.; Graham, D.; Baranska, M.; Majzner, K. Sensitive Detection and Identification Method of Erythrocyte-like Cells upon Doxorubicin Induced Differentiation with Vibrational Techniques. *Analytical Chemistry* **2025**, *97*, 16966–16974. PMID: 40726176, https://doi.org/10.1021/acs.analchem.5c02465.
2. Lyng, F.; Faoláin, E.; Conroy, J.; Meade, A.; Knief, P.; Duffy, B.; Hunter, M.; Byrne, J.; Kelehan, P.; Byrne, H. Vibrational spectroscopy for cervical cancer pathology, from biochemical analysis to diagnostic tool. *Experimental and Molecular Pathology* **2007**, *82*, 121–129. https://doi.org/10.1016/j.yexmp.2007.01.001.
3. Movasaghi, Z.; Rehman, S.; Rehman, I.U. Raman Spectroscopy of Biological Tissues. *Applied Spectroscopy Reviews* **2007**, *42*, 493–541. https://doi.org/10.1080/05704920701551530.
4. Sitarz, K.; Czamara, K.; Bialecka, J.; Klimek, M.; Zawilinska, B.; Szostek, S.; Kaczor, A. HPV Infection Significantly Accelerates Glycogen Metabolism in Cervical Cells with Large Nuclei: Raman Microscopic Study with Subcellular Resolution. *International Journal of Molecular Sciences* **2020**, *21*. https://doi.org/10.3390/ijms21082667.
5. Wiercigroch, E.; Szafraniec, E.; Czamara, K.; Pacia, M.Z.; Majzner, K.; Kochan, K.; Kaczor, A.; Baranska, M.; Malek, K. Raman and infrared spectroscopy of carbohydrates: A review. *Spectrochimica Acta Part A: Molecular and Biomolecular Spectroscopy* **2017**, *185*, 317–335. https://doi.org/10.1016/j.saa.2017.05.045.

6. Terán, M.; Ruiz, J.J.; Loza-Alvarez, P.; Masip, D.; Merino, D. Open Raman spectral library for biomolecule identification. *Chemometrics and Intelligent Laboratory Systems* **2025**, *264*, 105476. https://doi.org/10.1016/j.chemolab.2025.105476.
7. Angle, K.J.; Nowak, C.M.; Grassian, V.H. Organic acid evaporation kinetics from aqueous aerosols: implications for aerosol buffering capacity in the atmosphere. *Environ. Sci.: Atmos.* **2023**, *3*, 316–327. https://doi.org/10.1039/D2EA00092J.
8. Sikirzhytskaya, A.; Sikirzhytski, V.; Lednev, I.K. Raman spectroscopic signature of vaginal fluid and its potential application in forensic body fluid identification. *Forensic Science International* **2012**, *216*, 44–48. https://doi.org/10.1016/j.forsciint.2011.08.015.
9. Sitarz, K.; Czamara, K.; Bialecka, J.; Klimek, M.; Zawilinska, B.; Szostek, S.; Kaczor, A. HPV Infection Significantly Accelerates Glycogen Metabolism in Cervical Cells with Large Nuclei: Raman Microscopic Study with Subcellular Resolution. *International Journal of Molecular Sciences* **2020**, *21*. https://doi.org/10.3390/ijms21082667.
10. Konorov, S.O.; Schulze, H.G.; Piret, J.M.; Turner, R.F.B.; Blades, M.W. Evidence of marked glycogen variations in the characteristic Raman signatures of human embryonic stem cells. *Journal of Raman Spectroscopy* **2011**, *42*, 1135–1141. https://doi.org/10.1002/jrs.2829.
11. Olaetxea, I.; Valero, A.; Lopez, E.; Lafuente, H.; Izeta, A.; Jaunarena, I.; Seifert, A. Machine Learning-Assisted Raman Spectroscopy for pH and Lactate Sensing in Body Fluids. *Analytical Chemistry* **2020**, *92*, 13888–13895. PMID: 32985871, https://doi.org/10.1021/acs.analchem.0c02625.
12. Golparvar, A.; Boukhayma, A.; Loayza, T.; Caizzone, A.; Enz, C.; Carrara, S. Very Selective Detection of Low Physiopathological Glucose Levels by Spontaneous Raman Spectroscopy with Univariate Data Analysis. *BioNanoScience* **2021**, *11*, 871–877. https://doi.org/10.1007/s12668-021-00867-w.
13. De Gelder, J.; De Gussem, K.; Vandenabeele, P.; Moens, L. Reference database of Raman spectra of biological molecules. *Journal of Raman Spectroscopy* **2007**, *38*, 1133–1147. https://doi.org/10.1002/jrs.1734.
14. Golparvar, A.; Kim, J.; Boukhayma, A.; Briand, D.; Carrara, S. Highly accurate multimodal monitoring of lactate and urea in sweat by soft epidermal optofluidics with single-band Raman scattering. *Sensors and Actuators B: Chemical* **2023**, *387*, 133814. https://doi.org/10.1016/j.snb.2023.133814.
15. Ahlawat, S.; Kumar, N.; Uppal, A.; Kumar Gupta, P. Visible Raman excitation laser induced power and exposure dependent effects in red blood cells. *Journal of Biophotonics* **2017**, *10*, 415–422. https://doi.org/10.1002/jbio.201500325.
16. Grytsyk, N.; Boubegtiten-Fezoua, Z.; Javahiraly, N.; Omeis, F.; Devaux, E.; Hellwig, P. Surface-enhanced resonance Raman spectroscopy of heme proteins on a gold grid electrode. *Spectrochimica Acta Part A: Molecular and Biomolecular Spectroscopy* **2020**, *230*, 118081. https://doi.org/10.1016/j.saa.2020.118081.
17. Vlasov, A.V.; Maliar, N.L.; Bazhenov, S.V.; Nikelshparg, E.I.; Brazhe, N.A.; Vlasova, A.D.; Osipov, S.D.; Sudarev, V.V.; Ryzhykau, Y.L.; Bogorodskiy, A.O.; et al. Raman Scattering: From Structural Biology to Medical Applications. *Crystals* **2020**, *10*. https://doi.org/10.3390/cryst10010038.
18. Zhu, G.; Zhu, X.; Fan, Q.; Wan, X. Raman spectra of amino acids and their aqueous solutions. *Spectrochimica Acta Part A: Molecular and Biomolecular Spectroscopy* **2011**, *78*, 1187–1195. https://doi.org/10.1016/j.saa.2010.12.079.
19. Rimskaya, E.; Shelygina, S.; Timurzieva, A.; Saraeva, I.; Perevedentseva, E.; Melnik, N.; Kudrin, K.; Reshetov, D.; Kudryashov, S. Multispectral Raman Differentiation of Malignant Skin Neoplasms In Vitro: Search for Specific Biomarkers and Optimal Wavelengths. *International Journal of Molecular Sciences* **2023**, *24*. https://doi.org/10.3390/ijms241914748.
20. Synytsya, A.; Judexova, M.; Hoskovec, D.; Miskovicova, M.; Petruzelka, L. Raman spectroscopy at different excitation wavelengths (1064, 785 and 532 nm) as a tool for diagnosis of colon cancer. *Journal of Raman Spectroscopy* **2014**, *45*, 903–911. https://doi.org/10.1002/jrs.4581.
21. Bergholt, M.S.; Zheng, W.; Lin, K.; Huang, Z.; Ho, K.Y.; Yeoh, K.G.; Teh, M.; So, J.B.Y. Characterizing variability in in vivo Raman spectra of different anatomical locations in the upper gastrointestinal tract toward cancer detection. *Journal of Biomedical Optics* **2011**, *16*, 037003. https://doi.org/10.1117/1.3556723.
22. Shang, L.; Tang, J.; Wu, J.; Shang, H.; Huang, X.; Bao, Y.; Xu, Z.; Wang, H.; Yin, J. Polarized Micro-Raman Spectroscopy and 2D Convolutional Neural Network Applied to Structural Analysis and Discrimination of Breast Cancer. *Biosensors* **2023**, *13*. https://doi.org/10.3390/bios13010065.
23. Darvin, M.E.; Sterry, W.; Lademann, J.; Vergou, T. The Role of Carotenoids in Human Skin. *Molecules* **2011**, *16*, 10491–10506. https://doi.org/10.3390/molecules161210491.
24. Udensi, J.; Loughman, J.; Loskutova, E.; Byrne, H.J. Raman Spectroscopy of Carotenoid Compounds for Clinical Applications—A Review. *Molecules* **2022**, *27*. https://doi.org/10.3390/molecules27249017.
25. Tfaili, S.; Gobinet, C.; Josse, G.; Angiboust, J.F.; Manfait, M.; Piot, O. Confocal Raman microspectroscopy for skin characterization: a comparative study between human skin and pig skin. *Analyst* **2012**, *137*, 3673–3682. https://doi.org/10.1039/C2AN16292J.
26. Lin, D.; Lin, J.; Wu, Y.; Feng, S.; Li, Y.; Yu, Y.; Xi, G.; Zeng, H.; Chen, R. Investigation on the interactions of lymphoma cells with paclitaxel by Raman spectroscopy. *Spectroscopy* **2011**, *25*, 23–32. https://doi.org/10.1155/2011/701408.

27. Abramczyk, H.; Brozek-Pluska, B.; Kopec, M.; Surmacki, J.; Błaszczyk, M.; Radek, M. Redox Imbalance and Biochemical Changes in Cancer by Probing Redox-Sensitive Mitochondrial Cytochromes in Label-Free Visible Resonance Raman Imaging. *Cancers* **2021**, *13*, 960. https://doi.org/10.3390/cancers13050960.
28. Amaral, S.; Da Costa, R.; Wübbeling, F.; Redmann, K.; Schlatt, S. Raman micro-spectroscopy analysis of different sperm regions: a species comparison. *Molecular Human Reproduction* **2017**, *24*, 185–202. https://doi.org/10.1093/molehr/gax071.
29. Rygula, A.; Majzner, K.; Marzec, K.M.; Kaczor, A.; Pilarczyk, M.; Baranska, M. Raman spectroscopy of proteins: a review. *Journal of Raman Spectroscopy* **2013**, *44*, 1061–1076. https://doi.org/10.1002/jrs.4335.
30. Feng, X.; Moy, A.J.; Nguyen, H.T.M.; Zhang, J.; Fox, M.C.; Sebastian, K.R.; Reichenberg, J.S.; Markey, M.K.; Tunnell, J.W. Raman active components of skin cancer. *Biomedical Optics Express* **2017**, *8*, 2835. https://doi.org/10.1364/BOE.8.002835.
31. Czamara, K.; Majzner, K.; Pacia, M.Z.; Kochan, K.; Kaczor, A.; Baranska, M. Raman spectroscopy of lipids: a review. *Journal of Raman Spectroscopy* **2015**, *46*, 4–20. https://doi.org/10.1002/jrs.4607.
32. Silveira, L.; Sathaiah, S.; Zângaro, R.A.; Pacheco, M.T.T.; Chavantes, M.C.; Pasqualucci, C.A.G. Correlation between near-infrared Raman spectroscopy and the histopathological analysis of atherosclerosis in human coronary arteries. *Lasers in Surgery and Medicine* **2002**, *30*, 290–297. https://doi.org/10.1002/lsm.10053.
33. Huang, Z.; McWilliams, A.; Lui, H.; McLean, D.I.; Lam, S.; Zeng, H. Near-infrared Raman spectroscopy for optical diagnosis of lung cancer. *International Journal of Cancer* **2003**, *107*, 1047–1052. https://doi.org/10.1002/ijc.11500.
34. Gniadecka, M.; Wulf, H.C.; Nymark Mortensen, N.; Faurskov Nielsen, O.; Christensen, D.H. Diagnosis of Basal Cell Carcinoma by Raman Spectroscopy. *Journal of Raman Spectroscopy* **1997**, *28*, 125–129. https://doi.org/10.1002/(SICI)1097-4555(199702)28:2/3<125::AID-JRS65>3.0.CO;2-#.
35. Mahadevan-Jansen, A. Raman spectroscopy for the detection of cancers and precancers. *Journal of Biomedical Optics* **1996**, *1*, 31. https://doi.org/10.1117/12.227815.
36. ChemicalBook. Sodium Lactate (312-85-6). https://www.chemicalbook.com/SpectrumEN_312-85-6_Raman.htm. Accessed: 2025-11-06.

Disclaimer/Publisher's Note: The statements, opinions and data contained in all publications are solely those of the individual author(s) and contributor(s) and not of MDPI and/or the editor(s). MDPI and/or the editor(s) disclaim responsibility for any injury to people or property resulting from any ideas, methods, instructions or products referred to in the content.
